# Improving Bambara Groundnut Production: Insight Into the Role of Omics and Beneficial Bacteria

**DOI:** 10.3389/fpls.2022.836133

**Published:** 2022-03-02

**Authors:** Caroline Fadeke Ajilogba, Oluwaseyi Samuel Olanrewaju, Olubukola Oluranti Babalola

**Affiliations:** ^1^Food Security and Safety Focus Area, Faculty of Natural and Agricultural Science, North-West University, Mafikeng, South Africa; ^2^Division of Agrometeorology, Agricultural Research Council, Natural Resources and Engineering, Pretoria, South Africa

**Keywords:** Bambara groundnut, beneficial bacteria, food security, microbiome engineering, omics

## Abstract

With the rise in the world population, environmental hazards caused by chemical fertilizers, and a decrease in food supply due to global climate change, food security has become very pertinent. In addition, considerable parts of agriculture lands have been lost to urbanization. It has therefore been projected that at the present rate of population increase coupled with the other mentioned factors, available food will not be enough to feed the world. Hence, drastic approach is needed to improve agriculture output as well as human sustainability. Application of environmentally sustainable approach, such as the use of beneficial microbes, and improved breeding of underutilized legumes are one of the proposed sustainable ways of achieving food security. Microbiome-assisted breeding in underutilized legumes is an untapped area with great capabilities to improve food security. Furthermore, revolution in genomics adaptation to crop improvement has changed the approach from conventional breeding to more advanced genomic-assisted breeding on the host plant and its microbiome. The use of rhizobacteria is very important to improving crop yield, especially rhizobacteria from legumes like Bambara groundnut (BGN). BGN is an important legume in sub-Saharan Africa with high ability to tolerate drought and thrive well in marginalized soils. BGN and its interaction with various rhizobacteria in the soil could play a vital role in crop production and protection. This review focus on the importance of genomics application to BGN and its microbiome with the view of setting a potential blueprint for improved BGN breeding through integration of beneficial bacteria.

## Introduction

Today, there is an incisive reduction in crop production and diversity due to the inability to domesticate wild species, climate change impact, and urbanization (because of increasing population). For example, rice, wheat, and maize are the leading food crops in the world out of the thousands of the cultivable crop species available (Kumar et al., 2018). Furthermore, given the increasing world population, the production of sustainable food supplies will be a critical challenge in the 21st century. The world population is projected to cross 9 billion by 2050, indicating that food supplies must be doubled to meet the requirement of the expanding population (Laplaze et al., 2018). Beside increasing the quantity of food, improving quality is also critical to maintaining nutritive values. In addition to the mentioned problems, the use of chemical fertilizers in crop production has been a major cause of concern in their roles in increasing availability of chemical hazards in the environment. Hence, the need and advocation of an environmentally sustainable alternative to improving agricultural productivity has increased significantly.

Therefore, need for biodiversity in food crops and the incorporation of other lesser-known crops which are mostly referred to as underutilized/orphan crops, into the major food system is important now than ever. Various conventional and molecular breeding approaches have been employed to increase food production but there is still a very long way to go in meeting food demand. Molecular breeding methods have implemented the crop genome sequences as a key factor for understanding the processes (both physiological and biochemical) controlling plant traits and plant’s mode of responses to biotic and abiotic stresses. The rapid evolution of genome sequencing technologies has resulted in the generation of large data of plant genomes. This has created an opportunity for the application of this technology to crop improvement (Saima et al., 2017; Yuan et al., 2017) through various technologies. In the vastly improving area of life science technologies, new areas have merged for elucidating gene functions and metabolic pathways; “omics” technologies coupled with improved bioinformatics tools and databases. As our understanding of the key processes increases, it must be translated to researches in plant development and improved crop yield. Various next-generation-based technology has been developed to the generated large data sets. These protocols have been applied in genome-wide association studies (GWAS), quantitative linkage locus (QTLs) analysis, linkage mapping, genome selection, population genetics, marker-assisted breeding, genome editing, and SNP detections (Singh et al., 2011, 2017). All these protocols have been rightly optimized and modified for several model crops. A vast number of underutilized legumes have been proposed to help achieve food security especially in sub-Saharan Africa.

Legumes which are the most important food crops behind cereals belong to the family Leguminosae. They are important sources of proteins and minerals which makes them essential to poor people in underdeveloped communities most especially in Africa and Asia where the majority cannot afford meat and fish. Utilization of legumes in combatting malnutrition and food insecurity (Ojiewo et al., 2015; Mubaiwa et al., 2018; Olanrewaju et al., 2022) has been one of the focus of research in most developing countries. This focus outlines the visible potentials that need exploiting in these crops. Areas where they are majorly grown, include Nigeria, Senegal, Togo, Indonesia, Cameroun, India, and Cote d’Ivoire (Borget, 1992). They are classified as pulses (Bambara groundnut), oilseeds (Soybean and Groundnut), forage legumes (Winged bean), tuberous root (Yam bean), and food crops (Cowpea; Foyer et al., 2016; Mayes et al., 2019). Some are well known and extensively incorporated into the global food system while some are still relatively unknown and underutilized. Underutilization of these legumes can be because of a lack of knowledge/information on their uses and values. Among the underutilized legumes are African yam bean, Pigeon pea, and Bambara groundnut (BGN). Furthermore, a subset of these underutilized legumes perform better in marginal soils and under less favorable environmental conditions than their major crop counterparts (Olanrewaju et al., 2022). Hence, developing these subsets further for future agriculture makes a suitable and complementary approach to the continued use of major crops. This is particularly important given the expected negative impact of climate change on current major crop production systems and the gap between the current rate of genetic improvement of most major crops and the higher rates of food production required to be able to feed the predicted 9 billion in 2050 (Laplaze et al., 2018).

One of such underutilized crops Bambara groundnut [*Vigna subterranea* (L.) Verdc.], an indigenous African legume that thrives in marginalized soils where other crops do not thrive well (Halimi et al., 2019; Mayes et al., 2019; Olanrewaju et al., 2022). It is classified under the family *Fabaceae*, sub-family *Faboidea*, and the genus *Vigna* (Olanrewaju et al., 2022). There are two varieties known as the wild variety (*Vigna subterranea* var. spontanea) and the cultivated variety (*Vigna subterranea* var. subterranea; Yao et al., 2015). This legume seed crop was reported to originate from West Africa from the Bambara district near Timbuktu (Jideani and Diedericks, 2014) but is now widely grown in Africa, Malaysia, South, and Central America, some parts of Northern Australia, Sri Lanka, and Indonesia (Azam-Ali et al., 2001). Different places have their indigenous names for BGN for example in Malawi (Madagascar groundnut, Baffin pea, Voandzou, Indhlubu, underground bean, Nzama), Nigeria (Epa-Roro), South Africa (Jugo beans), and Zimbabwe (Nyimo beans; Emelike and Barber, 2018; Majola et al., 2021; Olanrewaju et al., 2022).

Its ability to fix nitrogen improves soil fertility and makes it useful in crop rotation and the possibility to be grown without the use of expensive chemicals and fertilizers (Babalola et al., 2017). Bearing in mind that chemicals and fertilizers are usually difficult to obtain in isolated areas, therefore, nitrogen fixation by BGN adds to its advantages to farmers especially in developing nations where it is cultivated. This crop has shown large diversity in its genetic resources for improvement and its richness in nutrient and mineral composition have been well-documented (Adebola et al., 2017; Atoyebi et al., 2017; Halimi et al., 2019; Valombola et al., 2019; Khan et al., 2020; Nyau et al., 2020; Adegboyega et al., 2021; Hlanga et al., 2021; Olanrewaju et al., 2021b).

**Figure 1:**
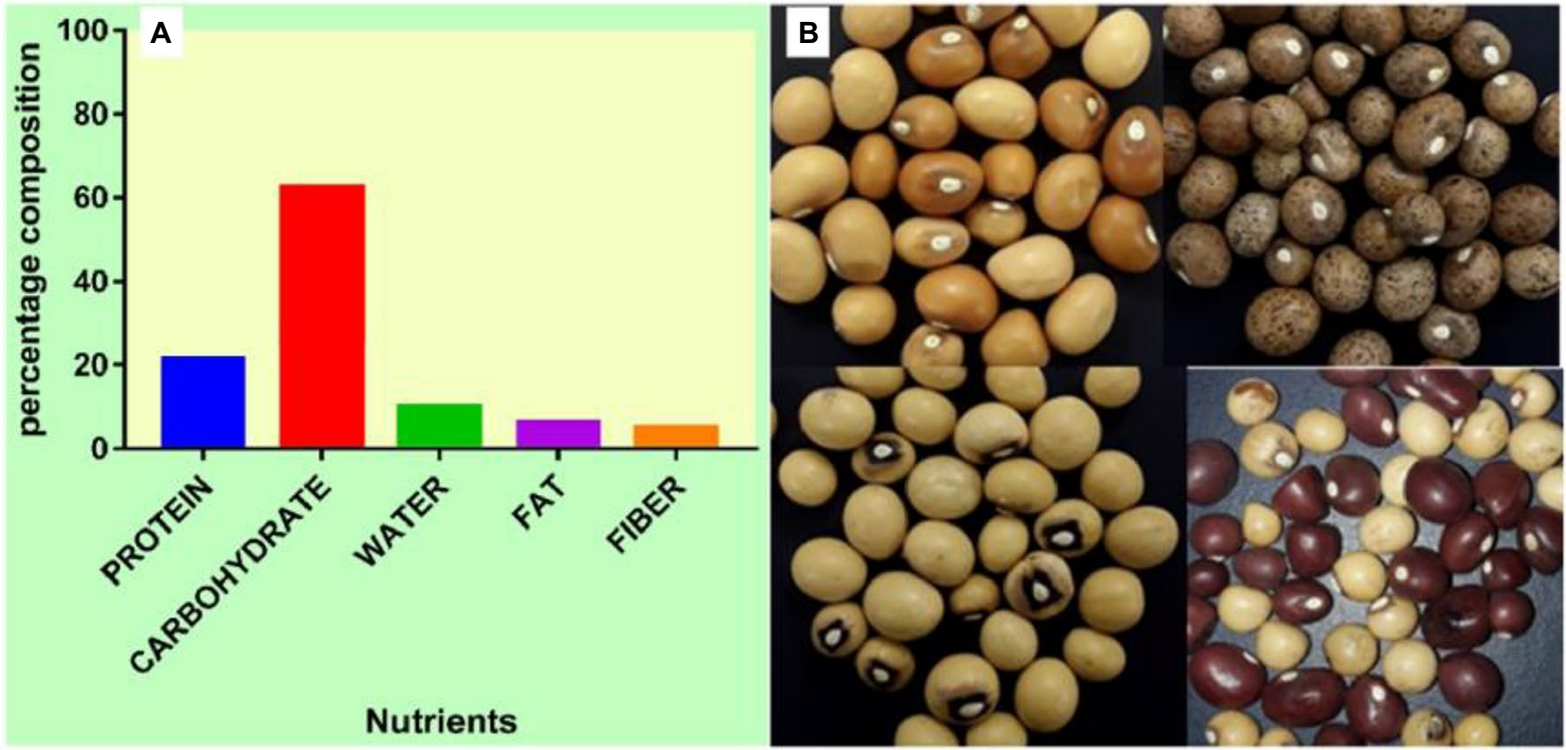
**(A)** Percentage nutrient composition of BGN. **(B)** Seeds of BGN showing variations in color, shape, and eye pattern.

Furthermore, BGN reportedly ticks the box for achieving food security crop (Massawe et al., 2016; Halimi et al., 2019; Olanrewaju et al., 2022). It is available, accessible, and affordable, and it is a source of income for farmers in sub-Sahara Africa due to its ability to tolerate drought and fix atmospheric nitrogen (Paliwal et al., 2020; Majola et al., 2021; Olanrewaju et al., 2021c). Furthermore, it cross-nodulates with nitrogen-fixing bacteria from other leguminous plants like cowpea (Laurette et al., 2015; Hassen et al., 2022) which further enhances its nitrogen-fixing ability. In sub-Saharan Africa, it is the third most eaten legume after groundnut (*Arachis hypogea*) and cowpea (*Vigna unguiculata*; Omoikhoje, 2008; Olukolu et al., 2012; Chai et al., 2017). It is nutritionally comparable to other legumes (Figure 1), such as soybeans, in the essential amino acids of lysine, methionine, and cysteine (Bamishaiye et al., 2011; Halimi et al., 2019). Compared to pigeon pea, lentils, and cowpea, BGN seed has a higher gross energy value (Hillocks et al., 2012). The protein of BGN seed competes favorably with other legumes, such as groundnut, cowpea, and pigeon pea, and it is found to be superior to the protein of other legumes (Halimi et al., 2019; Olanrewaju et al., 2022).

It can be eaten boiled or milled into flour before cooking. In Senegal, different concoctions made from the leaves, roots, and leaf sap have been used to treat infected wounds and abscesses, as an aphrodisiac, and to treat epilepsy, respectively, (Brink et al., 2006). The plant has been used by the Ibo tribe in Nigeria to treat venereal disease, while the seeds that were pounded and mixed with water were used to treat cataracts. Its leaves were used as fodder to feed animals (Brink et al., 2006).

Crop diversification through the inclusion of underutilized legumes into major crop system will enhance food availability, however, activities in soil have been shown to have a great impact in crop production and response to environmental stress. Soil contains myriads of microorganisms which function in promoting plant growth through direct or indirect activities. Out of these microorganisms, bacteria are the most documented, both beneficial and pathogenic. The beneficial bacteria, referred to as plant growth-promoting rhizobacteria/bacteria (PGPR/B; Olanrewaju et al., 2017), have been a breakthrough in food production. The bacterial-mediated activities in the rhizosphere include those beneficial to humans, such as the key role they play in the biogeochemical cycles of the main elements (carbon, nitrogen, and sulfur) and of trace elements (Iron, Nickel, and Mercury). These activities increase the involvement of bacteria in energy exchanges within the soil (Haichar et al., 2014). Bacteria also synthesize vitamins, auxins, enzymes, and other growth factors for plant growth promotion and disease suppression (Olanrewaju et al., 2017). Bacterial communities in the rhizosphere are influenced by the soil characteristics, type of host plant, and host developmental stages (Chaparro et al., 2014; Anzuay et al., 2021). BGN and other underutilized legumes are critical for achieving food security, especially in sub-Saharan Africa.

No established varieties of BGN which means that the crop is cultivated from local landraces instead of having different varieties. The ability of breeding programs to harness the advantages of the new life science technologies which apply various molecular markers in gaining a better understanding of BGN genetics is a great step in the right direction. Due to the importance of this crop, these technologies can improve farmer’s income through improved crop yield as well as improving both global and local food security.

With the issue of underdevelopment and climate changes, there is no better solution to combat the looming food scarcity than to look within. Therefore, the improvement of indigenous crops, such as BGN, will proffer a lasting solution in a continent like Africa. Furthermore, not much work has been done on the bacterial community from the rhizosphere of BGN and its interactions with the plant itself. Hence, this review aims to outline the importance of beneficial microbes in improving BGN production and the outline the use of omics techniques as an effective means to characterize BGN-beneficial microbe interaction mechanisms. This will provide a theoretical basis for improving BGN breeding through the combined application of omics and plant–microbe interactions to achieve food security. The findings should prove useful in exploring mechanisms of the relationship between BGN and associated microbial species.

## History of Bambara groundnut, Agronomy, and Morphology

The origin of BGN can be traced to Africa (Hillocks et al., 2012; Jideani and Jideani, 2021b; Soumare et al., 2021). Its history dates back to ancient Mali near Timbuctoo, from where the English name Bambara was derived from the Bambara tribe, even though they do not lay claim to the plant (Masindeni, 2006). Its center of origin can be traced to North Central and North Eastern Nigeria all the way to Northern Cameroon and the Central African Republic (Olukolu et al., 2012). Beyond Africa, it is seen to grow in other tropical nations like Greece, the Middle East, Malaysia, Indonesia, and, most especially, Brazil and Tropical America, where it is supposed that slaves must have helped to transport it to these nations (Brink et al., 2006).

It is an indigenous African crop that is common to many African countries, from Sudan in the North to South Africa in the South; from Kenya in the East to Nigeria in the West and even to Madagascar (Bamishaiye et al., 2011). It is one of the many underutilized and under-researched indigenous grain legumes. Female subsistence farmers in sub-Saharan Africa are the major growers of BGN (Mkandawire, 2007; Majola et al., 2021).

The annual production of BGN was estimated at 330,000 t in 1982 with about 50% coming from West Africa (William et al., 2016). In most of the semi-arid lands, yields from the farm pods vary between 650 and 850 kg ha^−1^ (Sangare, 2012). Since the crop is being produced at the subsistence level and not so much on a large scale, worldwide production figures have been difficult to collate but Zambia is still the most extensive producer (Opoku, 2010) while Nigeria, Burkina Faso, Niger, Mali, Ghana, Cote D’Ivoire and Chad are the major producers with only Burkina Faso, Chad, Mali, Niger and Senegal as the main exporting countries (Brink et al., 2006).

According to a report by Bamishaiye et al. (2011), BGN is a member of the family Fabaceae. It is a small plant-like groundnut which grows to a height of between 0.30–0.35 m. It is an intermediate plant with branched stems forming a bunch just above the ground. The plant grows as a small herb with compound leaves of three leaflets which are trifoliate and alternate from erect petioles. The peduncles of the leaves bear one or two flowers and are auxiliary branches from the stems. The stem begins to branch out very early after planting (Sangare, 2012). After fertilization, the flowers of BGN are pale yellow in color and they hang on the branching stems; these stems then grow downwards into the soil, taking the developing seed with it. The seeds form pods encasing seeds just below the ground in a similar fashion to peanuts. BGN pods are of different shapes, sizes, and colors, such as round, wrinkled, smooth, over 1.27 cm long, and white, cream, dark-brown, red, or black, and may be speckled or patterned with a combination of these colors, respectively (Figure 1). The roots with numerous nitrogen-fixing nodules grow from the short internodes of the stem to form a thick taproot with lateral roots developing as outgrowths toward the tip (Tweneboah, 2000). It is referred to as an autogamous plant (Baudoin and Mergeai, 2001). The structure of this plant shows that it helps to conserve space and more seeds can be planted on a small expanse of land without fear of low harvest. Its autogamy helps it to be available all through the year since it can be cultivated without the need for external pollination before flowering and seeding. Its availability throughout the year represents one of the core values of food security (Onwubiko et al., 2011).

## Importance of Bambara Groundnut Cultivation

BGN is cultivated for various reasons. It is known to have both agronomic and nutritional advantages.

### Agronomic Advantages of Bambara Groundnut

BGN is quite important in agriculture and a drought-tolerant crop (Masindeni, 2006; Babalola et al., 2017; Tan et al., 2020; Olanrewaju et al., 2021c; Soumare et al., 2021). It can perform well and have good crop yield on marginal soils and soils that have undergone water stress compared to other legumes (Brink et al., 2006). It grows well even in poor and infertile soils (Opoku, 2010; Olanrewaju et al., 2022). Because it tolerates poor soil, farmers with poor resources, especially with respect to purchasing fertilizer to increase yield, are encouraged to farm more with BGN. As a legume, BGN roots form a symbiotic association with root nodules of bacteria. This helps to increase the nitrogen content of the soil in the sense that the bacteria assimilate atmospheric nitrogen, trap it, and make it available to the plant in the soil (Babalola et al., 2017). This process in turn helps to increase soil fertility, which leads to increased crop yield (Masindeni, 2006). There are claims that it can reduce pests in the field (Ajayi and Lale, 2001; Udeh et al., 2020). It is however affected by storage pests which are the main cause of yield loss in most legume crops. These pests include cowpea weevil, bruchids, groundnut jassid, and brown leaf beetles (Majola et al., 2021).

### Nutritional Advantages of Bambara Groundnut

BGN is a complete food having different composition of carbohydrate, protein, and fat enough to serve as a balanced diet (Ijarotimi and Esho, 2009; Halimi et al., 2019; Tan et al., 2020; Majola et al., 2021; Olanrewaju et al., 2022). Further processing by fermentation also improves its nutritive and mineral components (Murevanhema and Jideani, 2013). Compared to pigeon pea, lentils, and cowpea, BGN’s seed has a higher gross energy value (Bamishaiye et al., 2011). The protein content is found to be of a higher quality (16–25%) compared to other legumes (Adegboyega et al., 2021; Hlanga et al., 2021). Its carbohydrate and fat composition are 65 and 6.5%, respectively, (Mazahib et al., 2013). Its fat content is higher than that of cowpea (1.0 ± 1.6%) and pigeon pea (1.2 ± 1.5%) but lower than that of groundnut (peanut; 45.3 ± 47.7%) with an estimate of between 5% (Sangare, 2012) and 6.3% (Omoikhoje, 2008). The composition of its protein is superior in essential amino acids and includes phenylalanine, lysine, valine, methionine, leucine, threonine, and isoleucine. Its fatty acid composition is also high in palmitic, linolenic, and linoleic acids (Halimi et al., 2019). The protein in BGN is rich in lysine and methionine comprising 6.6 and 1.3% of the total protein, respectively. It is also a rich source of iron, potassium, calcium, and fiber (Omoikhoje, 2008; Hillocks et al., 2012).

### Bambara Groundnut as Food

BGN is eaten when not matured by boiling it with salt and pepper. In many West African countries, it is consumed as a snack. It can also be made into flour when it is dry and mature because the seeds are hard (Tweneboah, 2000). The flour is used to prepare soup in East Africa with or without condiments while the flour has also been used to make bread in Zambia (Cook, 2017). The seeds can also be roasted, after which they are boiled, crushed, and eaten as a snack. Furthermore, the ground seeds can be used to make “akara” and “moinmoin” or the popular “okpa” in Nigeria (Emelike and Barber, 2018).

GIHOC cannery in Nsawam, Ghana, canned BGN seeds in gravy. Over 40, 000 cans of various sizes were prepared and made available throughout the year (Baudoin and Mergeai, 2001) and was comparable to Heinz baked beans even though its production declined due to competition with high yielding groundnut varieties and pest-resistant cowpea.

Vegetable milk extracted from BGN has been found to compete favorably with vegetable milk from soyabean, cowpea, and pigeon pea (Murevanhema and Jideani, 2013). The milk when properly processed has also been used as a weaning complementary food for children (Majola et al., 2021). The seeds were used as feed for poultry and piggery while its haulm and leaves which are rich in phosphorus and protein are used as fodder for cattle (Pui et al., 2021). BGN mixed with other leaf proteins has been used as an aquaculture feed with distinct growth in the fish (Adeparusi and Agbede, 2005).

### Medicinal Importance of Bambara Groundnut

Different preparations from BGN have been shown to have medicinal properties (Murevanhema and Jideani, 2013). Preparations from the leaves have been applied to infected wounds and abscess; extracts from the leaves have been applied to the eyes to cure epilepsy (Khan et al., 2021) while leaf extracts pounded with that of *Lanfana trifolia* L. have been used as an insecticide to wash livestock (Mkandawire, 2007). Venereal disease is treated by the Igbo tribe in Nigeria using the plant (Oluwole et al., 2021). Grounded Bambara seeds when mixed with water have been used to treat cataracts in Senegal and the root has been used as an aphrodisiac (Brink et al., 2006). Water from boiled maize and BGN when drunk is used by the Luo tribe of Kenya to treat diarrhea while in Botswana, the black seeded landraces have been used to treat impotency (Udeh et al., 2020). In South Africa, nausea was controlled in pregnant women who chewed and swallowed the seed (Jideani and Jideani, 2021a; Khan et al., 2021). In Ghana, pounded Bambara seeds have been used to treat skin rashes and the powder mixed with the meat of guinea fowl have been used as a treatment against diarrhea in children (Akpalu et al., 2013).

## Bambara Groundnut–Bacterial Interactions

**Figure 2:**
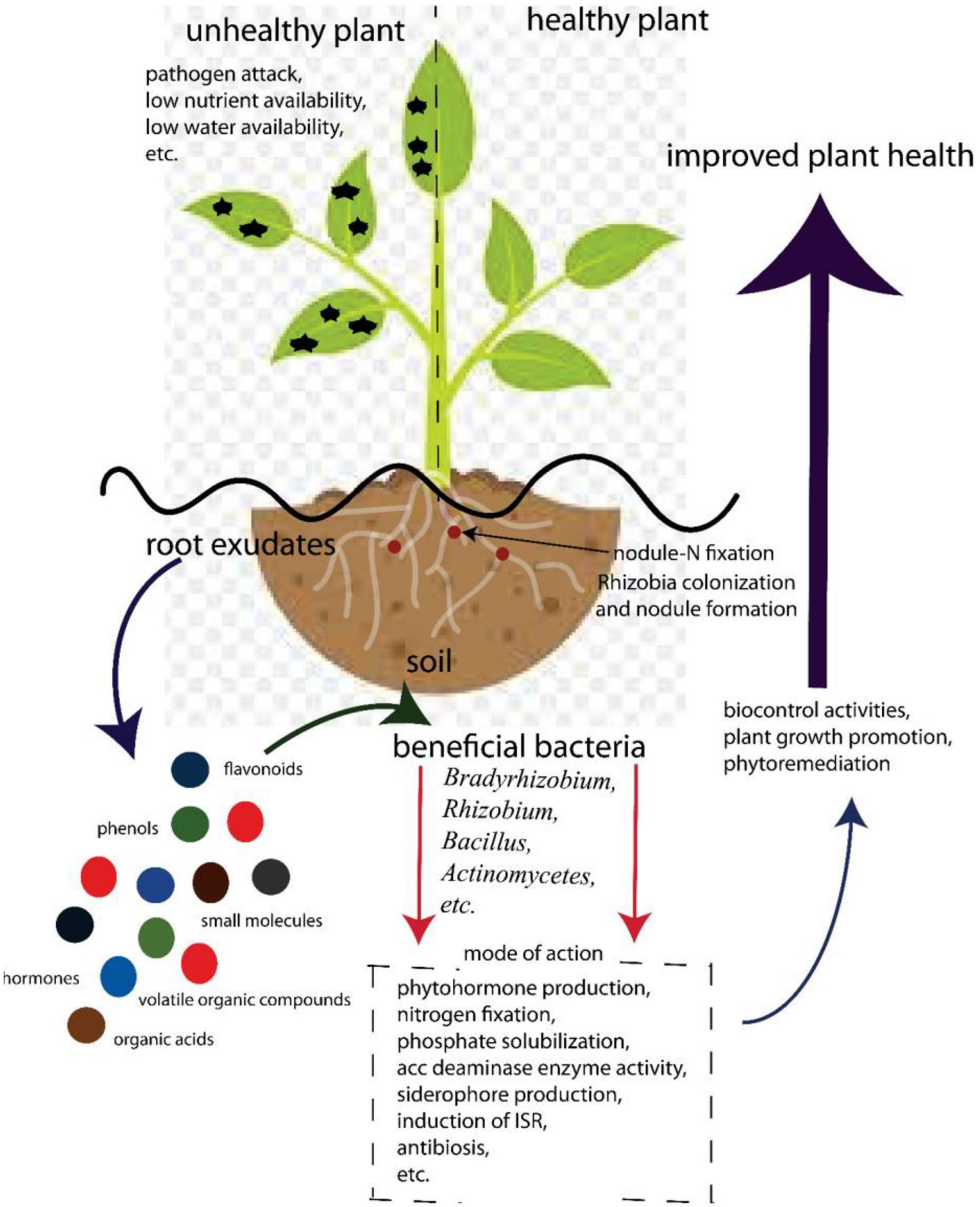
Beneficial associations involved in Bambara groundnut–bacterial interaction for food security.

The complexity of the rhizosphere is apparent in its role in plant health (Figure 2). Plants maintain this complex environment by secreting exudates into the rhizosphere (Olanrewaju et al., 2019). Plants secrete up to 40% of photosynthates that have access to roots in the rhizosphere (Berendsen et al., 2012). Because most of the soils are carbon deficient, these hot spots of carbon increase the microbial densities from 10 to 1,000 times, compared to bulk soil (Smalla et al., 2006). The increased concentration of microorganisms in the rhizosphere is due to the exchange of nutrients between the plant and the different taxa surrounding the root, which allows different types of associations. A number of factors influence the quantity and quality of root exudates, including plant species (Dennis et al., 2010), soil type (Berg and Smalla, 2009), plant developmental stage (Anzuay et al., 2021), pathogen attack (Olanrewaju et al., 2019), drought and heat stress (Ojuederie et al., 2019), and plant nutritional status (Carvalhais et al., 2011). If specific elements associated with the release of such exudates are better understood, novel approaches to enhancing beneficial microbial communities could be proposed.

Exudates released by plants contain phenolics, sugars, organic acids, and amino acids that attract both beneficial and non-beneficial microbes to the roots and serve as a source of carbon for these microbes (Figure 2). In exchange, beneficial microbes protect the plant against pathogens through well-documented mechanisms (Ahemad and Kibret, 2014; Olanrewaju et al., 2017; Backer et al., 2018). On the other hand, these carbon-containing compounds can also attract pathogens. Hence, they compete for nutrients, infect plants, and play a major role in structuring the rhizobiome.

Some plant species have been demonstrated to host-specific communities and attract protective microorganisms to suppress pathogens in the rhizosphere (Weinstein et al., 2021). Soil physical, chemical, and biological properties play an important role in the establishment of such plant–microbe interactions (Berendsen et al., 2012). Although pathogens can severely affect plant health, certain beneficial bacteria and fungi that also thrive in the rhizosphere, or inside plant tissues (endophytes) compete with these pathogens for space and nutrients; therefore exerting an antagonistic effect on them (Nihorimbere et al., 2011; Fadiji and Babalola, 2020; Verma et al., 2021).

PGPR grows in/on/or around root plant tissue and enhances plant growth, increases yield, protects plants against pathogens, and/or reduces biotic and abiotic stress (Trivedi et al., 2020; Ojuederie et al., 2021; Olanrewaju et al., 2021a). Growth promotion can be achieved directly by the interaction between the microbe and the host, as well as indirectly, due to antagonistic activities against plant pathogens. Direct interaction involves the production of phytohormones, which have been shown to inhibit or promote root growth, protect plants against biotic or abiotic stress, and improve nutrient acquisition by roots (Olanrewaju et al., 2017) and other direct-eliciting mechanisms. Because PGPR has the potential to replace chemical fertilizers and pesticides, it represents an environmentally sustainable alternative to increasing crop production and plant health. An interesting example of the role of microbial communities in plant nutrition and health is the interaction between rhizospheric fluorescent *Pseudomonas* and plants. Plants reduce soil iron (Fe) availability by acquiring iron and releasing exudates which attract the rhizospheric microbes that also utilize Fe. In Fe-stressed environments, siderophore-producing bacterial populations are enriched, which then suppress pathogens, such as fungi, for example, Oomycetes through competition for Fe. The plants, however, are able to utilize siderophores-bound iron, which enhances their growth (Lemanceau et al., 2013). This implies that the siderophore-producing microbes are able to produce the needed amount of Fe to the plants. Fe is an integral part of chlorophyll which is an important molecule in photosynthesis.

Another instance applied to plant disease suppression is the ability of resident microbiota in suppressive soils or compost to prevent pathogen infection (Hadar and Papadopoulou, 2012). In a soil suppressive to the fungal pathogen *Rhizoctonia solani*, Proteobacteria, Firmicutes, and Actinobacteria were prominent taxa found to be involved in disease suppression (Yin et al., 2013). There is also evidence to suggest that plants may use microbial communities to their own benefit to avoid infections (Mendes et al., 2011).

### Symbiotic Interactions in Bambara Groundnut Rhizosphere

Rhizobia species (*Rhizobium*, *Bradyrhizobium*, *Azorhizobium*, *Allorhizobium*, *Sinorhizobium*, and *Mesorhizobium*) have been known to suppress the growth of plant pathogens and form nodules in symbiotic relationships with legumes (Babalola et al., 2017; Grönemeyer and Reinhold-Hurek, 2018; Ibny et al., 2019). The symbiotic relationship also results in the production of nitrogen-rich soil. BGN forms nodules and fixes nitrogen in partnership with *Bradyrhizobium* strains (Ibny et al., 2019). Nodule formation is important in Bambara–microbe interaction; this process starts with the production of compounds, such as betaines, flavonoids, and aldonic acid as root exudates from the plant (Figure 2). These compounds signal to the rhizobia in a compatible relationship with the compounds. This in turn enhances the production of the nod gene that induces nodulation by interacting with the nodD protein of the cell wall of the rhizobia (Wang et al., 2018; Walker et al., 2020). The rhizobia react to this inducement by producing and releasing the lipo-chito-oligosaccharide Nod factors, which bring about morphological changes in the root hair of the legume. This leads to the formation of an infection thread and the development of nodules that finally enhance fixation of nitrogen (Wang et al., 2018). Nod factors produced by rhizobia are important in plant growth as they promote germination of seeds and the development of seedlings (Kidaj et al., 2012).

### Non-symbiotic Interactions in Bambara Groundnut Rhizosphere

Apart from the symbiotic relationship between plants and rhizobia, the production of phytohormones by these rhizobia, such as nod factors, riboflavin, and lipo-chito-oligosaccharide, can also stimulate plant growth and increase grain yield (Dakora, 2003). In addition, legumes generally produce phenolics (Tor-Roca et al., 2020) that help to suppress the activities of pathogens, make nutrients available to plants, and promote the growth of microorganisms with beneficial properties (Dakora, 2003).

Production of phenolic compounds mediates the production of nod gene inducers. The concentration of nod gene inducers regulate the production of nod factors around the root (Wang et al., 2018). When this is accumulated in the rhizosphere, it leads to biosynthesis of flavonoids, which also leads to an increased level of phytoalexin that is important for plant protection against pathogens (Dakora et al., 2015).

Rhizobia are also known to produce riboflavin (De Bruijn, 2015). It is a vitamin that is converted photochemically or by the actions of enzymes into lumichrome. This was evidenced in culture preparation from rhizobial cells. In its purified state, it was able to stimulate growth in maize, soybeans, and sorghum (Dakora et al., 2002). Rhizobia are important in suppressing the growth of pathogens of bacterial and fungal origins that infest sunflower, soybean, mungbean, and okra (Gopalakrishnan et al., 2015). Root exudates, such as phytosiderophores and organic acid anions, are important in making sure that minerals are available and circulate within the soil and agricultural systems (Dakora, 2003) that is the reason they are important in mixed cropping. Studies have shown that there was a continuous increase in yield and quality of seeds when BGN was inoculated with local strains of *Bradyrhizobia*. This is as a result of the increase in symbiotic nitrogen fixation activity of these microbes (Laurette et al., 2015).

## Potential of Genomics and Beneficial Microbes in BGN Production

Plants attract specific microbiomes from the soil and air to their various compartments including rhizosphere, endosphere, and phyllosphere. Intra- and inter-specific variations among plant species inferred significant differences in the microbiome composition of the different species. This indicates that host genetics play an important role in microbiome assembly and function. By the late 21st century, temperature simulation reports have predicted an increase and this will lead to reduction in yield of all crops (Anderson et al., 2020) including BGN. Advanced breeding for BGN yield improvement and climate-resilient trait is affected by low level of genetic diversity available for breeding programs. Genetic diversity of BGN has not been fully exploited nor used in breeding programs, and recent genomics advances have increased the possibility of characterizing large germplasm collections through genome-wide association analysis for the identification of single nucleotide polymorphism (SNP) markers which can further be used in genomics assisted breeding for improved traits at any season or BGN growth stage.

Application of genomics and beneficial bacterial in BGN breeding is a way forward to improve yield and quality traits of the crop especially with the expected climate change impact on the environment which is expected to affect crop production.

### Genomics for Improved BGN Production

Different markers are used for the genetic relationship and linkage mapping in BGN (Aliyu et al., 2016; Chai et al., 2017; Ho et al., 2017). The apparent use of the crop large germplasm for in improved breeding programs has caught the interest of breeders and geneticist. The genetic linkage map of BGN consisted of 11 linkage groups which originated from 223 markers (Ho et al., 2017). From the result obtained, it was observed that the linkage map showed synteny with their close relatives; mung bean, adzuki bean, and common bean genomes (Ho et al., 2017).

The Genetic Resource Centre (GRC) of the International Institute of Tropical Agriculture (IITA), Ibadan has the world’s largest collection of BGN accessions. Analysis of yield and yield stability, drought tolerance, nutritional and antinutritional contents have been carried out on the collection (Atoyebi et al., 2017; Adegboyega et al., 2021; Olanrewaju et al., 2021b,c). According to Paliwal et al. (2020), preliminary analysis showed significant variation in all accessions. This implies that there is a wide repertoire of genetic diversity and a great opportunity for genetic improvement in the crop. Furthermore, SNP genotyping has been applied paving the way for QTL analysis using GWAS. The aim is to develop core collections for future breeding research. So far, no GWAS study has been reported on BGN, hence, the importance of incorporating genomics into breeding programs cannot be over emphasized for this crop.

Furthermore, functional genomics increasingly allows the possibility of discovering new biocontrol, biostimulation, and biofertilization genes. Therefore, there is increased expectation in the exploitation of these genes for improved plant microbiome engineering. There is need for the implementation of global meta-omics approaches in elucidating the complex network of interactions in the plant microbiome. Integrative and comprehensive approach that combines multi-omics approaches, genetic engineering tools, synthetic biology, computational biology, bio-nanotechnology, and smart agricultural practices should be explored for the improvement of BGN production.

### Beneficial Microbes for Improved BGN Production

Beneficial effects [such as biotic and abiotic stress tolerance (Etesami and Maheshwari, 2018; Fazeli-Nasab and Sayyed, 2019; Ojuederie et al., 2019; Goswami and Suresh, 2020), increasing nutrient uptake (Dhawi et al., 2015; Turan et al., 2016; Olanrewaju et al., 2017; Abdiev et al., 2019), pathogen control (Olanrewaju and Babalola, 2019a,b; Castaldi et al., 2021; Dave et al., 2021), and growth promotion (Ajilogba et al., 2017; Valetti et al., 2018; Hakim et al., 2021; Olanrewaju et al., 2021a)] of bacterial on various plant hosts are well researched and very much understood. Ability to control and select for beneficial bacteria would proffer an avenue to improve these traits. However, impact of plant genotype on the bacterial microbiome must be well understood for proper implementation of the bacterial-traits-plant improvement. As said earlier, various species of BGN vary in their trait’s responses due to different large genetic diversities available in the BGN germplasm. Hence, mechanistic insight into specie-specific bacterial microbiome influence is needed for proper implementation of the bacterial microbiome in improving BGN production. Application of mechanistic insight of bacterial microbiome regulation on underutilized legumes like BGN is limited by the inability of reconciling decades of works on molecular plant microbe interactions with the diverse and constantly changing microbiota (Bulgarelli et al., 2012).

Furthermore, impact of microbe–microbe interactions on BGN must be considered when addressing the use of beneficial microbes on the plant. For example, beneficial bacteria are said to be beneficial because of the benefits they proffer on plants which include promotion of root growth by phytohormones, acquisition of iron by siderophores, nitrogen fixation, and degradation of ethylene through the action of ACC deaminase enzyme, and nodule formation (which is majorly on legumes). Considering the role played by rhizobium in forming nodules and fixing nitrogen for BGN, how does this affect the bacterial dynamics in BGN microbiome? How does BGN differentiate beneficial microbes from pathogens? What are the specific exudates specific for the various microbial species attracted to the plant’s biome? For example, auxin can suppress plant defense thereby enhancing plant’s susceptibility to pathogen attacks (Su et al., 2021) which is partly due to many pathogens also producing their own auxin and in the process alter host auxin biology (Chen et al., 2015). The feedback effects of the host processes on the microbiome and vice versa must be well defined to fully exploit the full potentials of beneficial bacteria in the plant biome.

The complexity of bacterial interactions clearly makes targeted plant breeding for microbiome engineering challenging. However, application of metagenomics and the discovery of microbial hubs allows for discovery of important beneficial bacteria that can serve as biomarkers for key traits at any stage of BGN growth. Absence or presence of such bacterial biomarkers associated with BGN can be utilized to establish beneficial bacterial community structures for the plant.

### Genomics-Enabled Approaches for Understanding BGN–Bacteria System Complexity

Engineering beneficial microbiota to improve BGN breeding requires improved linkage of the microbiota to the different BGN varieties. High-throughput amplicon sequencing has been productive in this aspect by linking microbial diversity to function in improving plant health (Brown et al., 2020). However, to understand how the environment, plant developmental stages, soil type, stress factors, and plant genotypes affect microbiome colonization, comprehensive characterization of BGN microbiome is needed. Through amplicon sequencing, especially 16S for bacteria microbiome, deep analysis of the BGN bacterial microbiota composition and functions can be predicted. Further application of long read metagenomics data with high-read sequencing will give a better depth and insight into the complexity and functions of BGN bacterial microbiota. Hence both approaches incorporated with BGN genome sequencing can be employed in targeted approaches to determine BGN genes that are involved in structuring the microbiome.

**Figure 3:**
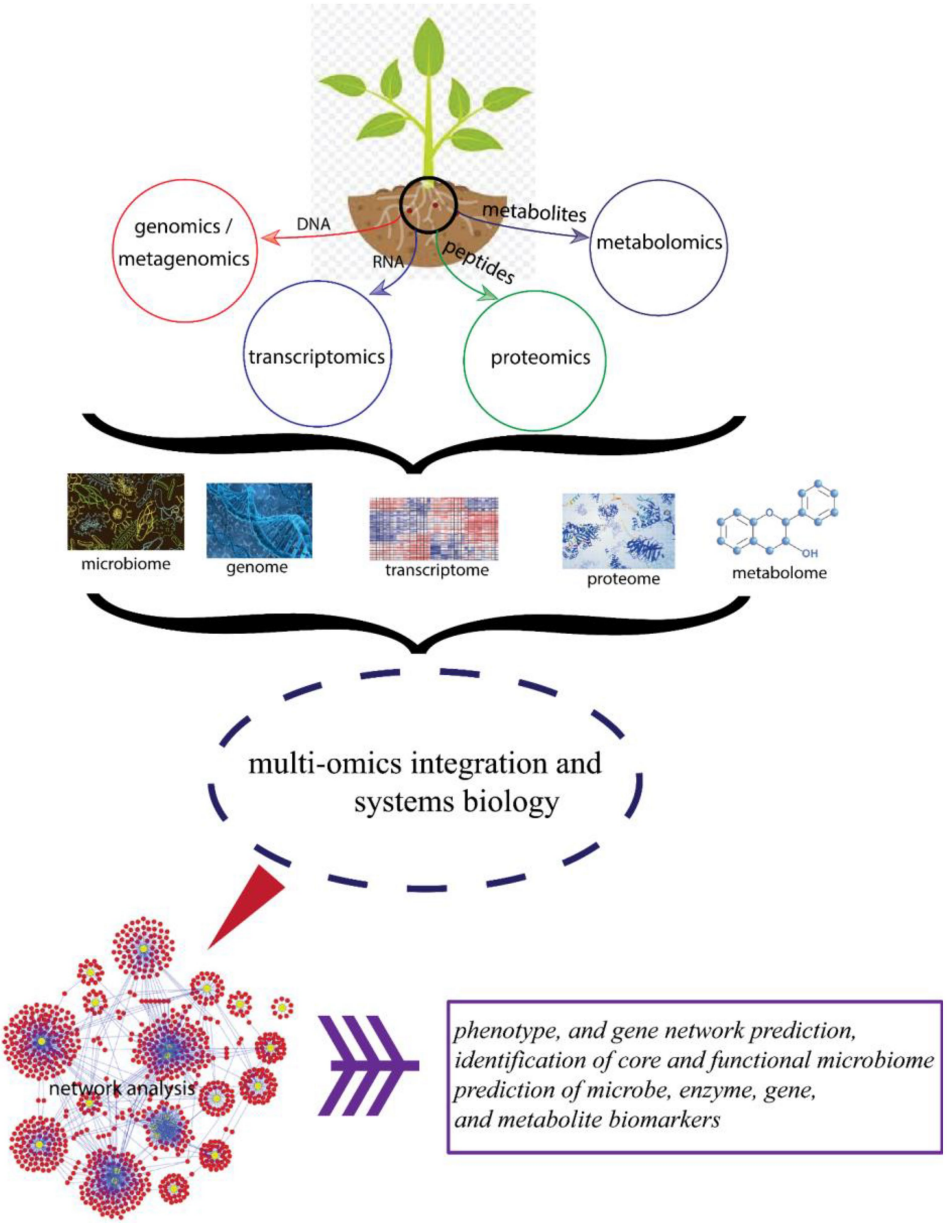
Schematic diagram showing different omics approaches and their beneficial effects toward improved plant health.

Furthermore, increasing availability of proteomics, transcriptomics, metagenomics, and metabolomics data will improve the identification of interaction and co-occurrence networks in BGN microbiome, thereby enabling the rapid discovery of microbiome interactions. This will result in improved breeding for important traits (Figure 3).

#### Transcriptomics for Studying BGN–Bacteria Associations

Transcriptomics investigates changes in expression of transcripts by using transcriptional regulators. Examples of such regulators include *AhNFR5A*, *AhNFR5B*, and *NodD* which regulate symbiotic processes and nodulation (Shu et al., 2020; Jiménez-Guerrero et al., 2021). In the expression of transcripts which occurs in response to microbe colonization, root exudates are key in these colonization activities. For example, flavonoids have been reported to be important in detecting protein-encoding genes in legumes during plant–microbe interactions in the process accounting for host specificity (Olanrewaju et al., 2019). Expression of protein-encoding genes in *S. meliloti* was regulated in *M. sativa* for the elucidation of gene interaction network (Chaudhary and Shukla, 2019).

The influence of plant growth-promoting microbes on the nodule and its bacteroid transcriptomes have been reported (Kelly et al., 2018; Lamouche et al., 2019). Researchers have employed laser-capture microdissection coupled with RNA sequencing for the total elucidation of expression patterns throughout the nodule compartment (Kusakin et al., 2021; Li et al., 2021). This technology is promising in fully understanding the gene expression patterns in nodules in response to microbe colonization.

Despite the availability of many studies addressing the transcriptional adaptations of microbes to their various hosts, many questions remain to be answered. For example, what is the molecular basis of host recognition? Specific role of signaling molecules, receptors, transcription factors, etc. Microbes might adapt their transcriptome in response to the host microbiome basically for survival in the biome. Therefore, does the transcriptomics modification of the microbiome by the expressing microbe have a causal effect on the genetic programming of the host? These and other yet unanswered questions may help in elucidating the mechanism of host and associated microbe transcriptomes for successful microbiome engineering application.

Comprehensive understanding of plant microbiome interactions will simplify the designing of microbial consortia capable of broad spectrum of plant growth-promoting abilities. Emerging technologies in microbiome functioning in addition to modern gene editing tools, such as CRISPR, RNAi, TALLEN, and so on, will make it possible to engineer microbial inoculants toward *in situ* microbiome engineering (Qiu et al., 2019; Trivedi et al., 2020).

Plants control diversity of their microbiome through a genetic network (Figure 3) in the process preventing dysbiosis. Hence, it is very possible with the availability of well-annotated genome, that BGN microbiome can be manipulated through genome editing of BGN plants with the recent advances in technologies, such as the CRISPR gene editing technology (Ramachandran and Bikard, 2019; Kumar and Dubey, 2020; Gao, 2021). With this, great opportunity lies ahead in comprehensive exploration of BGN–microbiome interactions to activate trait-specific members of its microbiota.

#### Proteomics for Studying BGN–Bacteria Association

Proteomics analysis technique improves our understanding of the regulation of protein expression. Studies at the proteome level can be used to identify cellular processes that occurs during interaction between plants and both beneficial bacteria as well as those induced by non-beneficial bacteria. In addition to identifying important proteins, proteomic studies can provide important information on genes that can be used in engineering plants toward biotic stress tolerance and improved plant growth. In simple organisms and complex organisms, proteomics has been a success in protein expression profiling. Proteomics was used to study plant–microbe interactions (Cooper et al., 2018), virulence factors in fungal pathogens (Rauwane et al., 2020), and plant’s responses to biotic and abiotic stress tolerance (Riviezzi et al., 2021). Proteomics has been applied in many studies involving legume–bacteria interactions (Table 1); however, no proteomic analysis has focused on BGN–bacteria association. This is partly due to the unavailability of a well-annotated reference genome.

**Table 1:** Omics studies of some legumes upon inoculation with beneficial bacteria.

Legume crops	Microbial inoculant	Inference from the studies	References
Transcriptomics			
*Lotus japonicus*	*M. loti* R7A, *Bradyrhizobium elkanii* USDA61, *Sinorhizobium fredii* HH103, *Pseudomonas syringae* pv. *tomato* DC3000, and *Ralstonia solanacearum* JS763	Correlation between bacteria compatibility and ability to induce responses in symbiosis and pathogenesis. Distinct transcriptome responses were observed in response to symbiotic and pathogenic bacteria.	Kelly et al., 2018
*Aeschynomene afraspera* and *Aeschynomene indica*	*Bradyrhizobium* sp. strain ORS285	In the study, dynamic role of oxygen and redox regulation of gene expression during nodule formation was suggested. The study invariably uncover the gene expression changes that accompany the transition of the bacterium from intracellular to differentiated bacteroids in the nodules.	Lamouche et al., 2019
*Arachis hypogaea*	*Bradyrhizobium* sp. strain SEMIA 6144	The study revealed a considerable overlap in expression profiles of most of the symbiotic genes between a crack-entry legume *A. hypogaea* and the IT-adapted model legumes, suggesting a functional conservation of most of the factors that govern the process of nitrogen-fixing symbiosis irrespective of the mode of rhizobial entry in their host plants.	Karmakar et al., 2019
*Medicago sativa*	*Ensifer meliloti*	From the study, alfalfa gene expression is strain-specific. Identified candidate genes underlying the specific interactions include *Medsa002106* and those encoding nodulins, NCR peptides, and proteins in the NBS-LRR family.	Kang et al., 2020
Proteomics			
*Medicago truncatula*	*Sinorhizobium meliloti* strain 2011	Characterized the proteome of *M. truncatula* root nodules in response to drought stress.	Larrainzar et al., 2007
*Glycine max*	*Bradyrhizobium elkanii*	Identification of differentially expressed proteins in soybean nodules under phosphorus deficiency.	Chen et al., 2011
*Medicago truncatula*	*Sinorhizobium medicae* WSM419 or *Sinorhizobium meliloti* strain 2011	Reduced level of leaf senescence during drought stress independent of the efficiency of the *Rhizobium* strain used.	Staudinger et al., 2016
*Pisum sativum*	*Rhizobium leguminosarum* bv. viciae RCAM	Proteomics analysis was used to probe seed metabolic differences related to simultaneous inoculation pea plants with rhizobia and AM fungi.	Mamontova et al., 2019
*Cicer arietinum*	*Bacillus amyloliquefacien*, *Bacillus subtilis*, *Lysinibacillus boronitolerans*, *Pseudomonas brassicacearum*	The study indicated that chickpea neutralizes an extensive range of functional responses to AHLs that may play important role in legume host–microbe interactions.	Saral et al., 2021
*Cajanus cajan*	*Sinorhizobium fredii* NGR234	Insights into the rhizobial proteins involved in the interaction between *Cajanus cajan*-Maize intercrop.	Vora et al., 2021
*Pisum sativum* and *Lens culinaris*	*Rhizobium leguminosarum* bv. viciae	Legume host affects the presence of multiple rhizobial proteins in nodule bacteroids.	Durán et al., 2021
Metabolomics			
*Phaseolus vulgaris*	*Fusarium oxysporum* f. sp. *phaseoli*	The study demonstrated alterations in metabolome concentrations after inoculation with the pathogen. As a result, significant changes in defense molecules were observed. Expectedly, pathogenesis-related genes were upregulated and flavonoid biosynthesis pathway was significantly enriched in response to the pathogen infection.	Chen et al., 2019
*Aeschynomene afraspera* and *Aeschynomene indica*	*Bradyrhizobium* sp. strain ORS285	47 metabolites that accumulated upon differentiation of the bacteroid in the nodules were reported when the plant was infected with the wild type or *bclA* mutant of the bacterium.	Lamouche et al., 2019
*Medicago truncatula*	*Arthrobacter agilis* UMCV2	The endophyte affects sugar composition in the plant leaves irrespective of the age of the leaf. Its impact is pronounced on the carbohydrate metabolism without affecting other metabolomes.	Ramírez-Ordorica et al., 2020
*Glycine max*	*Bradyrhizobium japonicum*	The study reported that the relative abundance of fatty acids, purines, and lipids was changed significantly in response to the symbiosis.	Agtuca et al., 2020

The availability of a well-annotated BGN reference genome will facilitate better understanding of the molecular mechanisms of interactions between BGN and its microbiome. Further application of ^15^N/^14^N metabolic protein labeling, one-dimensional gel electrophoresis (1D), LC-electrospray ionization quantitative time-of-flight (ESI-Q-TOF), 2-D electrophoresis/MALDI-TOF, and shotgun proteomic approaches will aid identification of the high complexity of proteins from BGN and its associated bacteria which may yield significant advances in BGN microbiome studies. A comprehensive study on legume–rhizobium symbiosis has been reviewed by Khatabi et al. (2019).

##### Proteomics and Beneficial Bacteria in BGN–Microbe Interaction

The most abundant form of nitrogen; dinitrogen gas (N_2_) is inert and cannot be accessed by plants. However, it can be converted through nitrogen fixation process by nitrogen-fixing bacteria (Babalola et al., 2017). These nitrogen-fixing bacteria encode the nitrogenase enzyme which catalyzes the reduction of N_2_ to ammonia according to the following reaction:


$$\begin{array}{l} N_{2}+{\mathrm{8e}}^{-}+{\mathrm{8H}}^{+}+16\mathrm{MgATP}\to {\mathrm{2NH}}_{3}+H_{2}+\\ 16\mathrm{MgADP}+16\;{\mathrm{PiN}}_{2}+{\mathrm{8e}}^{-}+{\mathrm{8H}}^{+}+16\mathrm{MgATP}\\ \to {\mathrm{2NH}}_{3}+H_{2}+16\mathrm{MgADP}+16\;\mathrm{Pi} \end{array}$$

Biological nitrogen fixation (BNF) is important in sustainable agriculture and mitigation of the environmental impacts posed by nitrogen fertilizers. Since the first report of BNF by plant-associated bacteria, there has been increased study of legume–bacteria symbiosis (Ferguson et al., 2019; Lindström and Mousavi, 2020; Schulte et al., 2021).

Glutamine synthetase, which uses ammonium as a substrate, catalyzes the conversion of glutamate to glutamine. This is followed by condensation of 2-oxoglutarate and glutamine to form glutamate. This process maintains glutamate homeostasis in nitrogen fixation. Hence, glutamine synthetase is believed to be of high importance in plant productivity with suggestions being made of it being a molecular marker for nitrogen uptake and utilization in plants (Swarbreck et al., 2011). Therefore, regulation of glutamine synthetase activities in BGN through proteomics could enhance knowledge for improved nitrogen efficiency and yield.

A symbiotic association for nodulation was found between the important model legume *Medicago truncatula* and *Sinorhizobium meliloti* (Larrainzar et al., 2007). In the study, proteomic approach was used to identify a total of 377 plant proteins in the nodules of *M. truncatula* using 2D-LC/MS/MS. Proteins reported are linked to nitrogen assimilation in nodules. Those involved in pathway of the synthesis of sulfur-containing amino acids were also reported. In another study by Nomura et al. (2010), the proteome of soybean-associating Bradyrhizobium japonicum was analyzed to evaluate time-dependent modulations in the proteome contents between 7 and 49 days of treatment.

Furthermore, recent advancement in visualization and modelling techniques allows identification of biomarker microbes in microbiome structure and function. Recent high-throughput culturing approaches can aid selection of significant microbes in microbial interactions which are related to better plant performance at various growth stages or in response to biotic and abiotic stresses. For example, Bez et al. (2022) identified several microbial groups showing negative correlation with *Dickeya* spp., a rice pathogen. These microbes can be targeted for improvements in controlling the pathogen. Applicable inoculants from these groups can be identified for specific and distinct abilities peculiar to each microbe in the group. Each microbe would exhibit distinct pathogen control mechanism and plant colonization; hence, large populations should be isolated and evaluated to obtain suitable microbes for further field trials.

In addition, gene expression analysis has aided determination of plant responses to bacteria colonization. However, the analyses of protein levels through proteomics technique are more essential for deciphering complex molecular mechanisms in plant–bacteria associations, especially as it applies to BGN–bacteria interactions.

Therefore, identification of other proteins involved in plant beneficial interactions through proteomics analysis of BGN–bacteria interaction is an avenue to harness the capacity of these beneficial bacteria on BGN yield. Furthermore, proteome studies not only provide insights into the activities of mRNA in the crosstalk between plants and beneficial bacteria, combined with metabolomics, it helps in elucidating the impact of the induced response on the plant phenotype.

#### Metabolomics for Studying BGN–Bacteria Associations

Metabolites are the end products of gene expressions. Metabolomics result directly correlates with the phenotype of the tissue or cell in study (Johnson et al., 2016; Figure 3). Hence, it is the link between genotypic effect on phenotypic responses. Root exudates in plants have been linked to the plant’s microbiome community. In Arabidopsis, for example, phenolic exudates positively correlates with the number of bacteria in the soil (Badri et al., 2013).

The mutualistic relationship between nitrogen-fixing rhizobia and soybean, an important legume, has been studied extensively. Soybeans’ dominant metabolites are of the classes isoflavones and saponins (Sugiyama, 2019), which act as signal molecules to attract nitrogen-fixing bacteria, such as *Bradyrhizobium*, *Rhizobium*, *Mesorhizobium*, and *Azorhizobium*. The importance of isoflavone in shaping the microbiome was revealed in the study by White et al. (2017) which showed the abundance of *Xanthomonodaceae* and *Comamonodaceae* in the rhizosphere of plants that can synthesize isoflavone over those that cannot.

Similarly, daidzein, an isoflavone, impacts the bacterial community in soybean root (Okutani et al., 2020). The findings in the study importantly report daidzein as specific in their recruitment of bacteria in the rhizosphere by attracting some and repelling others. Increased concentration of daidzein correlates with increased abundance of *Comamonadaceae* while rhizobia abundance decreases subsequently reducing the overall alpha diversity. This reduction in microbial diversity can be attributed to rhizobia not being able to efficiently use daidzein as a carbon source.

*Pseudomonas* sp. TLC6-6-5.4 and the endomycorrhizal mix of *Glomus intraradices*, *Glomus mosseae*, *Glomus aggregatum*, and *Glomus etunicatum* were inoculated in maize seedlings. After extraction and analysis of the metabolites by GC–MS (gas chromatography–mass spectrometry), upregulation of glyoxylate and dicarboxylate metabolism was observed (Dhawi et al., 2015). Single inoculation of PGPR alone or associated with endomycorrhizal mix increases the concentrations of mannitol, palmitic acid, lysine, stearic acid, and sucrose. Furthermore, metabolites produced showed correlation with nutrient uptake and biomass yield (Dhawi et al., 2015).

Metabolomics have been used to study mechanism of plant resistance to pathogens (Mhlongo et al., 2018; Zhu et al., 2018), drought stress (Mansour et al., 2021), production of antimicrobial compounds (Valette et al., 2020), and other focus which are presented in Table 1 as a result of inoculation with beneficial bacteria. These findings show that metabolomics is useful in screening BGN–bacteria associations for upregulation of important compounds that can be successfully regulated for BGN resistance to pathogens, drought tolerance, improved growth, yield, and nutrient components. Metabolomics, proteomics, and other omics applications will be useful in designing specific biofertilizer for BGN production.

## Conclusion

Application of beneficial microbes holds great promise in sustainable agriculture and achieving food security; however, reported successes have varied largely because of inefficient/poor colonization, unstable environmental condition, limited persistence especially in the rhizosphere as result of high competition for nutrients. Genome engineering and root colonization with large populations of beneficial microbes may help provide a solution. Advances in synthetic biology enables engineering and improvement of non-model microbes toward specific trait improvement. Upon inoculation of these engineered microbes, follow up on the ability of these microbes for successful colonization of the plant microbiome and their efficacy for growth promotion, pathogen control, and improving crop yield in pilot plots, greenhouses, and field demonstrations should be explored before commercial adoption. Multi-environment trials over lengthy periods and seasons should be carried out to evaluate the environmental impacts on the field treatments.

Omics technologies have improved our understanding of mechanisms involved in major biological systems. The application of these techniques coupled with beneficial microbes will advance our understanding of specific microbial adaptions to BGN and improve our knowledge in engineering a beneficial microbiome to improve its production. Hence, employing multi-omics approach to study BGN–bacteria association will be fruitful in the coming years.

## Author Contributions

All authors listed have made a substantial, direct, and intellectual contribution to the work and approved it for publication.

## Funding

OB is deeply appreciative of the 7 years of National Research Foundation (NRF) incentive funding (UID81192).

## Conflict of Interest

The authors declare that the research was conducted in the absence of any commercial or financial relationships that could be construed as a potential conflict of interest.

## Publisher’s Note

All claims expressed in this article are solely those of the authors and do not necessarily represent those of their affiliated organizations, or those of the publisher, the editors and the reviewers. Any product that may be evaluated in this article, or claim that may be made by its manufacturer, is not guaranteed or endorsed by the publisher.
